# Patient and public involvement in research: a review of practical resources for young investigators

**DOI:** 10.1186/s41927-023-00327-w

**Published:** 2023-03-09

**Authors:** Ashokan Arumugam, Lawrence Rick Phillips, Ann Moore, Senthil D. Kumaran, Kesava Kovanur Sampath, Filippo Migliorini, Nicola Maffulli, Bathri Narayanan Ranganadhababu, Fatma Hegazy, Angie Botto-van Bemden

**Affiliations:** 1 Department of Physiotherapy, College of Health Sciences, University of Sharjah, P.O. Box 27272, Sharjah, United Arab Emirates; 2 Neuromusculoskeletal Rehabilitation Research Group, RIMHS–Research Institute of Medical and Health Sciences, University of Sharjah, P.O. Box 27272, Sharjah, United Arab Emirates; 3 Sustainable Engineering Asset Management Research Group, RISE-Research Institute of Sciences and Engineering, University of Sharjah, P.O. Box 27272, Sharjah, United Arab Emirates; 4 Adjunct Faculty, Manipal College of Health Professions, Manipal Academy of Higher Education, Manipal, Karnataka India; 5 Patient Partner, www.RADiabetes.com, Indianapolis, IN USA; 6 Professor Emerita, School of Health Sciences, University of Brighton, 49 Darley Road, Eastbourne, BN20 7UR UK; 7 Department of Physiotherapy, Manipal College of Health Professions, Manipal Academy of Higher Education, Manipal, Karnataka India; 8 Centre for Health and Social Practice, Waikato Institute of Technology, Hamilton, New Zealand; 9 Department of Orthopaedic, Trauma, and Reconstructive Surgery, RWTH University Hospital, 52074 Aachen, Germany; 10 Department of Medicine, Surgery and Dentistry, University of Salerno, Baronissi, SA 84081 Italy; 11 School of Pharmacy and Bioengineering, Faculty of Medicine, Keele University, Stoke on Trent, ST4 7QB England, UK; 12 Queen Mary University of London, Barts and the London School of Medicine and Dentistry, Centre for Sports and Exercise Medicine, Mile End Hospital, London, E1 4DG England, UK; 13 MAPIMS - Melmaruvathur Adhiparasakthi Institute of Medical Sciences and Research, Melmaruvathur, Tamil Nadu India; 14 Global Patient Ambassador, Musculoskeletal Research International, Inc., Miami, FL USA; 15 Patient Partner, Holiday, FL USA; 16 EUPATI Fellow, Holiday, FL USA; 17 Clinical Research Experts, LLC., Tampa, FL USA

**Keywords:** Patient and public involvement, Patient engagement, Patient experience, Patient research partner, Guidance, How-to guide

## Abstract

Patient and public involvement (PPI) in every aspect of research will add valuable insights from patients’ experiences, help to explore barriers and facilitators to their compliance/adherence to assessment and treatment methods, bring meaningful outcomes that could meet their expectations, needs and preferences, reduce health care costs, and improve dissemination of research findings. It is essential to ensure competence of the research team by capacity building with available resources on PPI. This review summarizes practical resources for PPI in various stages of research projects—conception, co-creation, design (including qualitative or mixed methods), execution, implementation, feedback, authorship, acknowledgement and remuneration of patient research partners, and dissemination and communication of research findings with PPI. We have briefly summarized the recommendations and checklists, amongst others, for PPI in rheumatic and musculoskeletal research (e.g. the European Alliance of Associations for Rheumatology (EULAR) recommendations, the Core Outcome Measures in Effectiveness Trials (COMET) checklist and the Guidance for Reporting Involvement of Patients and the Public (GRIPP) checklist). Various tools that could be used to facilitate participation, communication and co-creation of research projects with PPI are highlighted in the review. We shed light on the opportunities and challenges for young investigators involving PPI in their research projects, and have summarized various resources that could be used to enhance PPI in various phases/aspects of research. A summary of web links to various tools and resources for PPI in various stages of research is provided in Additional file 1.

**Supplementary Information:**

The online version contains supplementary material available at 10.1186/s41927-023-00327-w.

## Background

Patient and public involvement (PPI) is essential throughout the life cycle of medical drugs and therapies development to ensure patients expectations, needs and preferences are met; subsequently, to improve patient adherence and most meaningful outcomes whilst reducing healthcare costs. Research programs/studies may fail to achieve meaningful patient impact and value if there is inadequate PPI preparation and integration. Clinical researchers helping with regulatory clinical trials often have direct access to patient research partners, or access to industry patient engagement departments; yet, basic science, biomedical, lab, translational, etc. researchers rarely have direct access to patients or professionals working in patient engagement and/or PPI to help identify and reach out to potential patient research partners to conceptualize research priorities and co-create suitable study design. Our aim is to prompt PPI access and action by providing a brief review of the most relevant PPI references/resources to refer to for practical guidance in musculoskeletal and rheumatology research for proper preparation to greatly increase the odds of successful and sustainable PPI in research.

PPI in medical/clinical research promotes "Nothing About Us Without Us” principles [1]. In the book "Nothing about us without us: Disability Oppression and Empowerment," Charlton highlights the rights of the differently abled community to participate in and advise about activities that impact its wellbeing. The principle started as a political concept demanding that people be consulted by their government to govern most effectively. This concept has been adopted in medical and clinical research with the increased advocacy/involvement by patients who want to ensure that research agendas best reflect outcomes which are most meaningful for disease management.

Today, funders are increasingly requiring researchers' to adopt PPI standards [2]. PPI in research is defined as "research being carried out 'with' or 'by' members of the public rather than 'to,' 'about' or 'for' them"[3]. This focus requires the researcher to consider PPI in all facets of the project.

PPI in research is implemented in practice by having discussions with patients as partners in various aspects of research (ideally all). Patients are often involved in surveys, interviews or focus groups to provide their views on the research topic, research question, methods and resources available. Exchanging the experiences of patient and public with researchers brings insights into why, how and when PPI in research could achieve the greatest benefits [4]. Given the challenges of data privacy or possible misuse in data-intensive projects, it would be beneficial to include patients in the administration of research projects (e.g., as members of a research project committee, research advisory board, or regular attendees of workshops) [5].

### Previously published points to consider PPI in research

Many authors have expounded on the benefits of PPI. Allison Worth identified the following reasons for a researcher to use PPI. Involving members of the public in research can help to ensure that: patient information sheets are legible, unequivocal, and easy to understand; research procedures are admissible to participants; volunteer enrolment and retention are optimal; and research findings are applicable to the patient experience" [6].

In addition, she found that, when the final interpretation of the study findings includes the "patient perspective", it improves the usefulness of the final research product [6]. Moreover, Shen et al. found that research using PPI was "more meaningful and culturally/socio-economically appropriate, identifying issues and details that researchers may not have been initially aware of" [7]. Honing the research question, prioritizing survey questions, and breaking the barriers to research has proven that PPI is economical and can prevent missteps that might have derailed the research process [7, 8].

PPI improves dissemination of the resultant data [5, 8, 9]. The key to having PPI members involved in sharing research results is that they have substantial interaction with the research team throughout all stages of the process to ensure comprehension and knowledge transfer [7, 8]. Proper PPI preparation ensures team competence and elicits capacity building.

Most researchers are happy to increase public awareness of study findings and dissemination of actionable insight at study completion. PPI members can effectively share the opportunity of co-presenting results at conferences [10], discussing concerns with the public [8], and co-appearing in the media [10].

Notably, prior to beginning any research project, the research team must first discern which gaps are most impactful whilst being feasible to address in the research setting and timeframe - this cannot be done without planning PPI to share the patients’ lived experience and what matters most. When patients are involved, there is the potential to broaden the pool of ideas about what is important to be researched. In the end, a more extensive collection of ideas about what should be asked can save time, money, and resources [7, 8]. In addition, it can allow a more meaningful outcome.

### How do we make research patient friendly?

Sir Mark Walport has stated that "Science isn't finished until it's communicated. The communication to wider audiences is part of the job of being a scientist, and so how you communicate is absolutely vital" [11]. The job of communicating our research to the public is more than simply publishing a scientific paper. Researchers must make a conscientious effort to engage patients/public early to most effectively co-create communications, determining together the best methods of communicating research to raise awareness of research opportunities and value as well as sharing research findings and actionable insights.

There are many tips for doing this, from blog posts to research articles as well as video interviews between researchers and patients. Importantly, PPI in research must be fit-for-purpose, understanding that each study will be unique. There are no one size fits all templates, yet guidance principles are generalizable and tools may be translational across therapeutic areas.

For example, following a large study concerning children's heart surgery [12], the organization Sense About Science [13] published a list of five best practices for researchers to engage with the public and patient stakeholders [14]:

1. Scoping—Determine what is being said about the research topic. Think about what others misconstrue about the issue.
2. Involve people—Define who is interested in your research and will likely want to hear about it.
3. Plan—Define how information is communicated about your topic. Find the hot spots where people gather to discuss the issue. Do others discuss in blogs, public meetings, or small group meetings? That might be a pivotal point in the findings.
4. Develop material—Ask your public and patient team members to help select the appropriate material.
5. Disseminate the material—meet, present, seek public interviews, and create blogs about results. At the same time, listen to the audience to refine the approaches you have taken to disseminate the information.

The above is a broad outline, and others suggest more practical steps that fit within that framework:

1. Explain it to your audience—do not supply too much or too little detail when discussing your research. Let people know what is important to them, and do so in a language they understand [15].
2. Make your research accessible to others—this might mean writing a blog or placing it on a website such as ResearchGate. It might also mean using Twitter, Facebook, or LinkedIn, then cross-referencing that place on any material passed out [15].
3. Give public talks—be willing to engage the public and patients in a two-way dialogue. Also, be ready to share by talking to small and large public groups [15].
4. Pay attention to the summary—making the summary more readable may be the best way to engage people. Use plain/lay language, so that the public and patients will have a good idea of the purpose and outcome in summary [16].
5. Use captions—for example in video content [16].
6. Choose accuracy over clarity—zeroing in on one act or outcome might exaggerate the purpose of the research. Be sure to paint the larger picture and the specific research in question [16].
7. Choose pictures—understandable pictures can communicate much more information than a column of data. When possible, present data with visual representation (e.g. an infographic) [17]. However, if you use pictures, be sure and use Alt text to communicate with those who may have visual difficulty [16].
8. Focus on the big trend—scientists might understand the difference between the big trend and a variant piece of data that establishes the micro-study. However, the public shall likely not. Be sure and highlight the big trends [17].

These steps can significantly improve how the findings are communicated and understood by all stakeholders. They might even assist in finding new interested audiences. For additional information and resources to facilitate PPI and patient engagement principles, please refer to the PPI Frameworks, Guidelines, Principles, Toolkits subheading in Tools and Resources table for more detailed information.

### Recommendations and checklists for patient involvement in rheumatic and musculoskeletal research

Approval for involving patient and public must be sought from the relevant ethics committee [18, 19]. The ethics committee verifies that the safety, integrity and rights of the participants in a research study are safeguarded, to provide opinions and create training opportunities on the ethical aspects of practice and research in biomedical sciences. At a national level, each country has recommendations and checklists for patient involvement in clinical research [20–24]. These guidelines aim to promote the efficiency of the clinical research, standardizing criteria and recommendations, and stimulating innovation and research. Guidelines aim to produce a favourable environment to conduct clinical research, through the harmonization of the rules and processes of evaluation, at the same time guaranteeing the highest standards for the safety of participants and transparency.

Several supervisory boards elaborate recommendations and checklists for patient involvement in clinical research. At global level, recommendations and checklists for patient involvement in clinical research have been published. In 2009, the European Alliance of Associations for Rheumatology (EULAR formerly the European League Against Rheumatism) initiated the development of recommendations for the inclusion of patient representatives, promoting standardized operational procedure for developing recommendations [25]. EULAR aggregates all European rheumatology societies, and is aimed at research, prevention, therapy, and rehabilitation of rheumatic diseases. It represents the patients, doctors, and scientific societies that deal with it. In 2011, EULAR published their recommendations to enable successful inclusion of the patient perspective EULAR funded scientific researches [25]. A patient research partner (PRP) [26] is defined as “partner with a relevant disease who operate as active research team members on an equal basis with professional researchers, adding the benefit of their experiential knowledge to any phase of the project” [25]. Moreover, a checklist for the recruitment of patients in research projects was put forward. This checklist offers a practical guidance for patient involvement in research projects. The checklist focused on eight endpoints: (1) the role of patient research partners, (2) phase of involvement, (3) the recommended number, (4) recruitment, (5) selection, (6) support, (7) training and (8) acknowledgement (Table 1) [25].

**Table 1:** The European Alliance of Associations for Rheumatology (EULAR) recommendations

Sections	Statement
Role	Participation of patient research partners is strongly recommended for clinical research projects and for the development of recommendations and guidelines, and should be considered for all other research projects
Phases of research	Participation of patient research partners should be considered in all phases of the project to provide experiential knowledge, with the aim of improving the relevance, quality and validity of the research process
Recommended number	A minimum of two patient research partners should be involved in each project
Recruitment	Identification of potential patient research partners should be supported by a clear description of expected contributions
Selection	The selection process of patient research partners should take into account communication skills, motivation and constructive assertiveness in a team setting
Support	The principal investigator must facilitate and encourage the contribution of patient research partners, and consider their specific needs
Training	The principal investigator must ensure that patient research partners receive information and training appropriate to their roles
Acknowledgement	The contribution of patient research partners to projects should be appropriately recognised, including co-authorship when eligible

The international Core Outcome Measures in Effectiveness Trials (COMET) Initiative seeks to promote the development and uptake of agreed standardized endpoints in clinical research, known as “Core Outcome Sets” (COS) (http://www.comet-initiative.org/). COS represent the minimum that should be measured and reported in all clinical trials of a specific condition. Patient and public input into core outcome set development is essential to ensure the use of patient-important outcomes in research. COMET established the People and Patient Participation Involvement & Engagement (PoPPIE) Working Group to support COS researchers internationally in developing COS with patients. Patients can participate in COS studies, for example, in surveys or consensus meetings. They can also be involved in COS studies, helping to design, oversee and disseminate studies. PoPPIE produced a PPI checklist for international COS developers to use when they involve patients.

Researchers newer to PPI may be encouraged to review the Guidance for Reporting Involvement of Patients and the Public (GRIPP) checklist, in advance, before conceiving and co-designing studies incorporating PPI as this may further elicit ideas and considerations for patient/public involvement when planning a research project. The GRIPP checklist has been developed to report data of patients involved in clinical researches [27, 28].  The updated version has both short and long forms (GRIPP2-long form [LF] and GRIPP2-short form [SF]) respectively [29]. The GRIPP-2 represents the first international evidence based, consensus informed guidance aimed to improve the quality, transparency, and consistency data of involved patients in research, along with a collaborative involvement of patients as research partners at all stages in the research [29]. The GRIPP checklist is summarized in Table 2.

**Table 2:** The Guidance for Reporting Involvement of Patients and the Public (GRIPP) checklist

Sections	Endpoints
*Section 1: Abstract of paper*	
1a: Aim	Report the aim of the study
1b: Methods	Describe the methods used by which patients and the public were involved
1c: Results	Report the impacts and outcomes of PPI in the study
1d: Conclusions	Summarise the main conclusions of the study
1e: Keywords	Include PPI, “patient and public involvement,” or alternative terms as keywords
*Section 2: Background to paper*	
2a: Definition	Report the definition of PPI used in the study and how it links to comparable studies
2b: Theoretical underpinnings	Report the theoretical rationale and any theoretical influences relating to PPI in the study
2c: Concepts and theory development	Report any conceptual or theoretical models, or influences, used in the study
*Section 3: Aims of paper*	
3: Aim	Report the aim of the study
*Section 4: Methods of paper*	
4a: Design	Provide a clear description of methods by which patients and the public were involved
4b: People involved	Provide a description of patients, carers, and the public involved with the PPI activity in the study
4c: Stages of involvement	Report on how PPI is used at different stages of the study
4d: Level or nature of involvement	Report the level or nature of PPI used at various stages of the study
*Section 5: Capture or measurement of PPI impact*	
5a: Qualitative evidence of impact	If applicable, report the methods used to qualitatively explore the impact of PPI in the study
5b: Quantitative evidence of impact	If applicable, report the methods used to quantitatively measure or assess the impact of PPI
5c: Robustness of measure	If applicable, report the rigor of the method used to capture or measure the impact of PPI
*Section 6: Economic assessment*	
6: Economic assessment	If applicable, report the method used for an economic assessment of PPI
*Section 7: Study results*	
7a: Outcomes of PPI	Report the results of PPI in the study, including both positive and negative outcomes
7b: Impacts of PPI	Report the positive and negative impacts that PPI has had on the research, the individuals involved (including patients and researchers), and wider impacts
7c: Context of PPI	Report the influence of any contextual factors that enabled or hindered the process or impact of PPI
7d: Process of PPI	Report the influence of any process factors, that enabled or hindered the impact of PPI
7ei: Theory development	Report any conceptual or theoretical development in PPI that have emerged
7eii: Theory development	Report evaluation of theoretical models, if any
7f: Measurement	If applicable, report all aspects of instrument development and testing (e.g., validity, reliability, feasibility, acceptability, responsiveness, interpretability, appropriateness, precision)
7g: Economic assessment	Report any information on the costs or benefit of PPI
*Section 8: Discussion and conclusions*	
8a: Outcomes	Comment on how PPI influenced the study overall. Describe positive and negative effects
8b: Impacts	Comment on the different impacts of PPI identified in this study and how they contribute to new knowledge
8c: Definition	Comment on the definition of PPI used (reported in the Background section) and whether or not you would suggest any changes
8d: Theoretical underpinnings	Comment on any way your study adds to the theoretical development of PPI
8e: Context	Comment on how context factors influenced PPI in the study
8f: Process	Comment on how process factors influenced PPI in the study
8g: Measurement and capture of PPI impact	If applicable, comment on how well PPI impact was evaluated or measured in the study
8h: Economic assessment	If applicable, discuss any aspects of the economic cost or benefit of PPI, particularly any suggestions for future economic modelling
8i: Reflections/critical perspective	Comment critically on the study, reflecting on the things that went well and those that did not, so that others can learn from this study

The Outcome Measures in Rheumatology conferences (OMERACT) have also proposed guidelines (and have identified facilitators) for patients/public to actively participate in conferences [30]. A key facilitator is to ensure that patient participation is an integral part of the conference vision so the patient perspectives are effectively included. For ﻿additional information and resources to facilitate PPI and patient engagement principles, please refer to the PPI Frameworks, Guidelines, Principles, Toolkits subheading in Tools and Resources Additional file 1 for more detailed information.

### What tools have already been developed to facilitate participation, communication and co-creation?

PPI in research is all about conducting research ‘with’ or ‘by’ members of the public, and has been recommended and supported widely [25, 26]. Conventionally, patients are often used in the initial stage and/or at the end stage of research. However, recently a few principles have been found essential in PPI such as equity, respect, trust, empowerment, role clarity and clarity on expectations, and understanding these may facilitate patient involvement through all stages of research planning and conduct.

Involvement can mean consultation, collaboration, or consumer-led research. In terms of the degree of involvement, patients/consumers can be involved as an object/respondent, advisor, interviewer/moderator, research partner and research principal [26]. The last two roles, patients as research partners and research principals, have progressively become more important. Patients involvement has gained momentum in the last decade, with patients identifying and prioritizing topics, reviewing grant applications, analyzing and interpreting data, and disseminating findings. The Department of Health (INVOLVE) unit in the UK has produced guidelines for both researchers and consumers, specific tools/checklists that facilitate or evaluate public participation in research [2].The earliest evidence for developing such a tool was a consensus study by Telford et al. [31]. To simplify the practical application of a patient (public/consumer) involvement tool, Hewlett et al. [32] proposed the “FIRST” (**F**acilitate [inclusion and contribution of patient partners], **I**dentify [projects, patients, roles], **R**espect [contribution and confidentiality of patient partners],  **S**upport [communication and working with patient partners] and **T**raining [with/of patient partners]) solution.

The PPI tools may help patient and public participants to consider their involvement as a partner in research in the following aspects: (i) to facilitate the patients’ inclusion as partners in the research and their ability to contribute to the study; (ii) to identify the projects from the point of patients and partners from the point of researchers; (iii) for the researchers to respect partners’ contributions and for partners to respect confidential information; (iv) to support the patient partners’ ability to communicate and work with the researcher and their group; (v) to train patient partners in terms of research methods and processes to develop some understanding of the process among the patient partners [32].

The EULAR has performed extensive work on PPI in research. The EULAR group has highlighted the role of patients as partners in developing patient reported outcome measures (PROs) [33]. This is important, as many PROs have been developed by health professionals and researchers with little or no participation of patients. Even though if patients were involved, they mostly had a passive role as a participant or respondent (equating to ‘consultation’ in participation ladder). Active patient participation is feasible and possible in the development of PROs, as demonstrated by a case study example of co-developing a PRO for psoriatic arthritis [33].

On the other hand, Stewart and Liabo [34] proposed an alternative model to the hierarchy model. This model places research at the centre, and acknowledges that researchers and patients may have difference expertise which contribute to and improve research relevance and quality. Therefore, relevant rigorous research requires inputs from patients/public, researchers and policy makers. This framework is simplistic, and ascertains involvement of consumers by asking the question “what expertise is needed for a study and when?” [34].

Patient/Public involvement in research collaboration is facilitated through various patient/participant-centric initiatives, using web-based information tools. These tools offer various modes to facilitate patient’s engagement and communication in PCI such as—(i) matchmaking; (ii) direct to consumer (DTC); (iii) dynamic negotiation; (iv) citizen science. *‘Matchmaking’ tools* facilitate active communication between willing participants who provide their personal information and researchers who identify the eligible participants using the information provided by the participants. Few examples of matchmaking tools are—ResearchMatch.org, TrialX.com, and EmergingMed.com. *DTC tools* help the researcher to identify the participant directly through offering some health benefits to them. For example, providing people with knowledge about their unique genotype and phenotype that may help them make informed decisions about their own health. Web-based DTC genetic testing companies like 23andMe.com and deCODEme.com aim to allow and motivate identity perception for individuals. To administer a multitude of participant preferences, such as personal access, a tool for active negotiation between participants and researchers to give people more levels of choice and control is required. Under personal access, patients can upload their health-related information and gain control over both their health records and the stakeholders that the data are accessible to. Such tools allow researchers to identify suitable participants. When participants offer researchers personal access, those researchers can view these private records and use the information for pertinent research initiatives [35].

Patient-Focused Drug development (PFDD) meetings are the formal meetings led either by Food and Drug Administration (FDA led PFDD meetings) or externally (EL-PFDD meetings) between patients, patients’ representatives/advocates/caregivers and FDA, clinicians, academicians, researchers in order to gain insights about patients’ experiences, perspectives, needs and priorities. Two panel discussion followed by an open discussion are conducted to facilitate patient participation and communication. Especially in the second panel discussion, patients are asked about their consideration on participating in the clinical trials [36].

Tools that encourage conversation and provide clarity regarding roles and expectations may be useful for PPI in research [2]. The "involvement matrix" use a participatory research methodology in which meaningful conversations between patients and researchers are made possible. The proposed research project's phases are also included in the involvement matrix (horizontal alignment), in addition to the function of involvement (vertical alignment). Five separate roles—a listener, co-thinker, adviser, collaborator, and decision maker—have been recognised [2], despite the matrix including three phases of research (preparation, execution, and implementation). The engagement matrix enables researchers to categorize and quantify patient roles in the progression of research studies. Patients might specify their preferred role when speaking with the researcher. The participation matrix is likely one of the best available tools for facilitating PPI in research.

Patient-powered research networks (PPRNs) are virtual channels managed by patients, patients’ representatives/advocates/caregivers and clinicians, academicians, researchers to gather health and clinical data and to use later in research. The emphasis is on gathering authentic data and employing patient-centered outcomes [37]. Examples of PPRNs include PCORnet (https://pcornet.org/), PatientsLikeMe (https://www.patientslikeme.com/), the Accelerated Cure Project (https://www.acceleratedcure.org/), the European Patients’ Academy on Therapeutic Innovation (EUPATI; https://eupati.eu/), etc.

### What are the qualitative research approaches to explore patients’ experiences of PPI and involve patients in research?

Qualitative research and PPI are considered an important component of developing and delivering public health interventions. The Patient Engagement for Medicines Development (PFMD) recently created “how-to guides” for PPI, including quantitative, qualitative and mixed methods approaches. The experiences and beliefs of the patients, carers, health professionals, and researchers conducting the study must be understood. Therefore, qualitative research is often the way towards PPI. Broadly, qualitative research includes different methods which mainly address queries relating to “why?”, “how?”, and “for whom?”. This type of research aims to document information and responses of participants in their own settings, trying to make sense of, or elucidate, phenomena pertaining to the actual meanings that participants bring to them [38].

All qualitative approaches such as in-depth interviews, focus group discussions, and participant observations can be applied to explore patients’ experiences of PPI to facilitate their involvement in research. However, there are a few differences in the way qualitative approaches are utilized in PPI. They are as follows: (i) the intention of qualitative approaches in PPI is to inform the public and gain insights form the public about research; (ii) the aim is to get first hand perspective about research implementation process at all levels; (iii) methodological variations including optional ethical approval, opportunistic sampling of participants through personal or web-based informatics tools. Overall, PPI focuses on improving the research process by involving the public and patients. [39].

Qualitative approaches utilize mixed method deigns in PPI to bring researcher, clinicians and patient/public together in order to discuss and share their ideas, understand patient/public experiences. Conversation though the whole process is often captured informally using for example meeting notes and may be recorded as a proof of the discussion and not necessary for analysis. Decision making in PPI happens in “real time”. The output of the discussion in PPI will be used to facilitate the research that is relatable and acceptable to the public and patients. [39].

To make sure that interventions are relevant, practical, and interesting for the PPI users, the Person-Based Approach (PBA) employs qualitative research at each level of development and execution [40]. The PBA for intervention/therapy development adapts and incorporates techniques from qualitative, mixed-methods, and user-centered design. This method of producing interventions offers a thorough comprehension of the opinions and encounters of intervention users as well as the environments in which they engage in behavioral change [40]. Hoven et al. used a qualitative study methodology to investigate the experiences of PRPs and research team involved in a co-creative sustainbale partnership in cancer research. Eleven PRPs and six researchers participated in semi-structured interviews through telephone, and five main domains were identified: reasons for engaging in a long-term cooperation, advantages of participation, strategies to improve the research, success factors and problems, and ways to improve [41]. Since PPI involves collaborative work among patient partners and researchers, and requires strong discussion between them, it is imperative that qualitative research methods are used across all the stages of patient involvement in the research. Mixed methods approach, using both quantitative and qualitative methods, as those described in the PFMD guidance, are at present considered best.

### Continuous learning and quality improvement of PPI research experience

Patient Engagement Quality Guidance tool (version 2) was developed by patients and those experienced in the medicine-development and feedback process [42]. The tool can reflect the quality and effect of the PPI research projects and the associated advantages they provide the stakeholders involved. The tool can be used for planning a PPI project, assessing the project, or as a feedback tool to identify the strengths and limitations of the projects which would help in planning future projects. It contains seven quality measures that incorporates the basic description of the project, the quality of PE in the project, the analysis of results and outcomes, and the message gained from the completed projects. This tool includes the following seven patient engagement quality criteria to plan, develop and evaluate the quality of PPI in research projects:

1. Shared purpose,
2. Respect and accessibility,
3. Representativeness of stakeholders,
4. Roles and responsibilities,
5. Capacity and capability for engagement,
6. Transparency in communication and documentation, and
7. Continuity and sustainability.

This guidance tool complies with the requirements of all stakeholders involved in PPI research projects [42].

The International Association for Public Participation (IAP^2^) aims to foster the practice of PPI in relation to all stakeholders affecting the public interest in all nations. This PPI framework aims to help public participation to reach better decisions, including the interests and concerns of all involved stakeholders and fulfill the requirements of the decision-making entity. The IAP^2^ Australasia quality assurance standard endorsed in 2015 for community and stakeholder engagement includes: defining the problem; agreeing on the purpose/context; knowing participation level; identifying stakeholders and developing relationship; describing project requirements; developing, approving and executing an engagement plan; evaluating, reviewing and providing feedback; and monitoring and documenting the evidence [43]. For additional information and resources to evaluate and/or improve PPI quality, please refer to the PPI Quality subheading in Tools and Resources Additional file 1 for more detailed information.

### Guidance on authorship/acknowledgement of patient partners in research (publications)

Authorship for PRPs is one step in demonstrating authentic, equitable partnership from the start versus acknowledgement which often reveals that the public and/or patients were added as an afterthought to fulfill requirements and/or content improvements requested by the journal guidelines, reviewers, editors, funders, or regulatory agencies. Authorship demonstrates accountability and responsibility for the published work and provides credit and important implications academically, socially and financially. The role of authors and non-author contributors is defined by the International Committee of Medical Journal Editors (ICMJE). The ICMJE recommends that authorship be based on the following four criteria:

1. Substantial contributions to the conception or design of the study or data collection, analysis or interpretation;
2. Writing the study or critical appraisal for important intellectual content;
3. Approval of the final version;
4. Accountability for all aspects of the study.

All designated authors must fulfill all four of the aforementioned ICMJE requirements to be included as an author; similarly, all those meeting the four criteria are to be included as authors [44]. PRPs are challenged in fulfilling all four criteria when researchers, or principal investigators have not involved patients at project, research, or study conception prompting opportunities to contribute to study design, acquisition, analysis and/or data interpretation. Researchers must have the strategic forethought to involve PRPs early as independent collaborators, or co-investigators at project conception; thus, they must proactively ensure that opportunities exist for patients to contribute as authors*.* Ideally, published work will also include the GRIPP-LF evaluation to further demonstrate PRP involvement from the beginning, or the GRIPP-SF if it was an afterthought [29]. Notably, including patients from the start of research helps with study conceptualization during which PRPs may offer novel insights on most impactful, meaningful aspects and concepts to address remaining gaps in knowledge about a condition, or illness, intervention and management options, and/or policy changes needed to diagnose and intervene earlier and prevent progression [45]. Research contributors not meeting the four ICMJE authorship criteria are instead acknowledged.

Notably, the GRIPP-SF or LF and/or lay language summaries, also referred to as plain language summaries, are now a requirement of many journals, funding, governing authorities, federally regulated clinical trials (U.S. FDA, European Medicines Agency [EMA], Health Technology [HTA], National Institute for Health and Care Research [NIHR], The Patient-Centered Outcomes Research Institute [PCORI]) and remain encouraged by others (The Canadian Institutes of Health Research [CIHR], National Institutes of Health [NIH]). For additional information and resources to facilitate PRP authorship and publications, please refer to authorship subheading in Tools & Resources Additional file 1 for more detailed information.

### Remuneration for patient research partners

A patient participant is not a patient representative and a patient representative is not a PRP—each provides a unique contribution to medical/clinical research. A patient participant, or study participant, may be reimbursed for their time and completion of experimental study procedures within the specified study timeframe. A patient representative may share their individual and/or representative community’s condition, or illness experience for a specific project. A PRP helps as a collaborative partner from the beginning, sharing insights and wisdom based on lived experiences, accounting for diverse experiences across the condition, or illness spectrum informing research priorities, care pathways and policies and how best to meet patient expectations, needs and most importantly preferences with multi-modal management, optimizing patient empowerment to self-manage. PRPs strengthen the quality and success of local, national and international research initiatives as a contributing partner of the research team [46–49].

True public or patient research partnership may not exist unless PRPs are considered as part of the research group and included from the beginning on the project billing analysis, or costs just as the principal investigator, co-investigators, research assistants, clinical research coordinators, etc. Patients with knowledge and/or experience of a specific disease, disorder, or illness provide an authentic accounting of what it is to live with it, interventions explored, healthcare delivery, and management dynamics, impacts on them and those in their social circle, as well as what their priorities are to manage their needs and quality of life, and how management is dynamic, changing throughout the continuum. The role of the PRP is applicable throughout the empirical research circle [50]. It is wisest to include public, or PRPs, as equal and valued partners just as other members of the research team are included on the individuals’ responsibilities research role/delegation form as well as the research administration billing forms. Many patients are willing to volunteer without remuneration; yet equity, diversity and inclusive input is best achieved with PRPs as part of the remunerated research team. Equity for patients as research partners is no longer merely encouraged, yet fit-for-purpose patient research partnership is required.

Many models of PPI describe varying levels of involvement for the research team to consider their fit-for-purpose PPI. PCORI provides a model capturing various levels of PRP engagement and their relationship to compensation levels [51, 52]. In 2015, the Change Foundation created a decision tool to help decide whether to pay their patient engagement participants or not (see Additional file 1 with a link to the online tool posted under a Creative Commons license). The tool applies to PPI with a fixed amount of time and not that which entails ongoing tasks, governance roles, paid advocacy, or contributions of professional expertise; yet it is applicable for most institutional or organizational research. It measures time, equity, vulnerable group status, challenges, accountability, positive impact, access, and other forms of recognition. Researchers may consider using this tool or at minimum make note of its categories, scores, etc., to make an informed PRP payment decision on a fit-for-purpose basis [53].

PPI frameworks identify numerous opportunities for PRP, including involvement in priority-setting, participation in governance committees, consulting on research design and knowledge translation activities, etc. Notably, involvement levels may change accordingly throughout the research phases/process. Payment of PRPs should reflect expertise level, time commitment, responsibility, type of work involved, and the extent of participation considered. Fit-for-purpose assistance and affability are provided to PRPs to ensure their valuable contribution to discussions and subsequent decisions. Such frameworks provide secure conditions that facilitate genuine discussions, intercultural competence, practice, and education. Assistance also signifies monetary compensation for their participation [53].

The CIHR Strategy for Patient-Oriented Research (SPOR) principles relate to persons offering services and therefore differs from the assistance provided to a patient organization for a relevant project (i.e., helping communication, supporting organizing a conference, etc.). Explaining these principles are out of the scope of this review, and are explained further by National Health Council (NHC) and Clinical Trials and Transformation Initiative (CTTI) Patient Advocacy Organization (PAO) framework.

SPOR Principles note that patients have the right to remuneration and that the level of remuneration should be equitable, optimal, and suitable for the services offered without exceeding the market value. SPOR principles also note that researchers, institutions, or entities must be on a par in the way they remunerate PRPs, and that PRPs have the right to refuse remuneration. The nature, amount, and details of compensation should reflect the extent of PRP involvement.

Compensation may vary based on the specific details of research responsibilities and should allow for some flexibility. All remuneration, irrespective of source, needs to subjected to relevant tax laws and regulations in a country. The appropriate tax authorities may need to be consulted to ensure compliance [51, 52].

Understandably, it may be convenient to recruit nearby colleagues or retired, financially comfortable and healthy PPI contributors for researchers with time demands to meet milestones, whilst also being held to ethical standards and governance boundaries. This type of convenience sampling results from lack of patient prioritizing, strategic forethought and sufficient funding. This ‘convenience sampling’ is neither equitable nor capable of delivering the diversity of views that would be expected of an ethical and pragmatic process whether borne of insufficient funding, lacking infrastructure, researcher laziness, individual bias, or misguided logic. This practice is particularly problematic, and the need for change is well-documented [54]. PRPs could be included early in the planning of research projects.

The preparedness and capability of expert and novice researchers engaging PRPs will require initiative to create a suitable environment for facilitating PPI and these efforts must be coordinated by the research team and related to the organizational infrastructure. For additional information and resources to facilitate PRP remuneration, please refer to the Patient Engagement Plan/Agreements/Contracts and Payment/Remuneration subheadings in Tools and Resources Additional file 1 for more detailed information.

## Conclusion

**Fig. 1:**
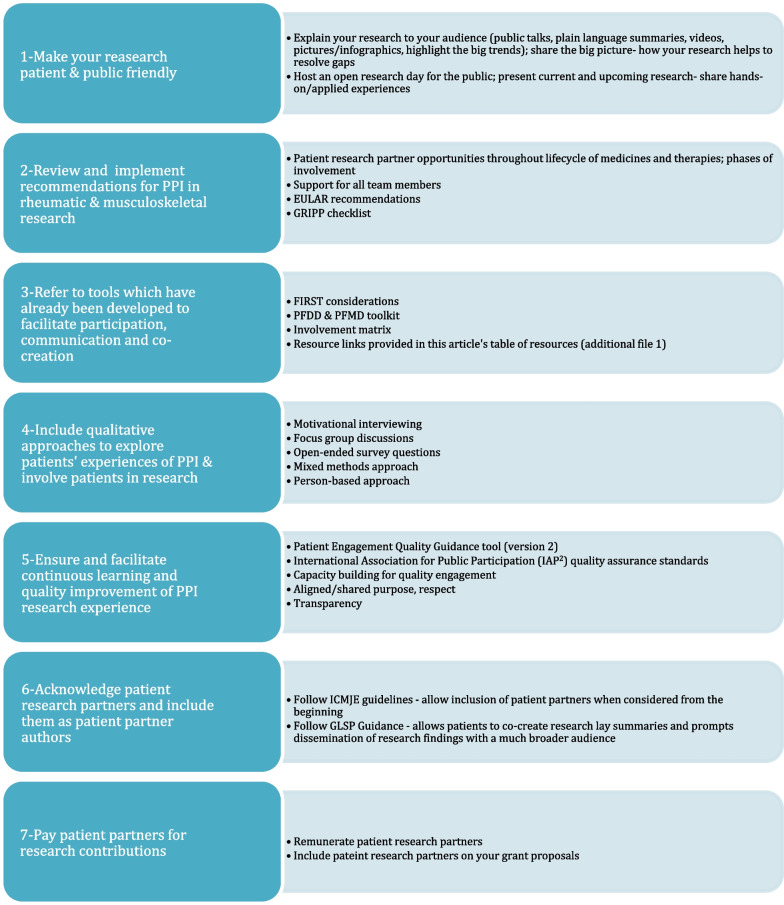
Seven practical ways to get started with patient and public involvement (PPI) in research. *EULAR* European League Against Rheumatism, *FIRST* Facilitate, Identify, Respect, Support and Training, *GLSP* Good Lay Summary Practice, *GRIPP* The Guidance for Reporting Involvement of Patients and the Public, *ICMJE* International Committee of Medical Journal Editors, *PFDD* Patient-Focused Drug development, *PFMD* Patient Engagement for Medicines Development

There are several challenges in PPI in research. Firstly, difficulty with access to terminology, meetings and training along with communication challenges could affect the active involvement and contribution of PRPs. Secondly, change in the relationship between patient and researcher from traditional clinician-patient relationship into researcher-patient partner relationship could lead to exposure to unintended confidential information. Thirdly, uninformed assumptions of researchers about patient partners such as having inadequate knowledge and inadequate contribution could lead to tokenism. Lastly, apprehension of taking a new role as a research partner, inexperience with the technical terms, unclear role definition, and concerns regarding the ability to contribute among patients could affect the active involvement and contribution of patients as a partner in the research []. Importantly, challenges create PPI opportunities for researchers and PRPs to co-create meaningful studies and/or research programs by setting research priorities together, developing protocol designs and research partnerships that are fit for purpose, improving recruitment and research awareness, team competence and capacity building, sharing of results and actionable insights, etc. Successfully realizing these PPI research opportunities will require a shift in mindset for most researchers and capacity building for all stakeholders—commitment to PPI competencies and investment in organizational infrastructure is imperative. This review summarizes the best practices, guidelines and checklists focusing on PPI. Figure 1 depicts the practical ways to get started with PPI in research. Please refer to the Additional file 1 for many valuable links to comprehensive PPI resources that could not be included in this review.

## Supplementary Information

**Additional file 1:** A summary of web links to various tools and resources for PPI in various stages of research.

## Data Availability

Not applicable.

## Abbreviations

CIHR: Canadian Institutes of Health Research
COMET: Core Outcome Measures in Effectiveness Trials
COS: Core Outcome Set
CTTI: Clinical Trials and Transformation Initiative
DTC: Direct-To-Consumer
EMA: European MedicinesAagency
EULAR: European League Against Rheumatism (European Alliance of Associations for Rheumatology)
EUPATI: European Patients' Academy on Therapeutic Innovation
FDA: Food and Drug Administration
FIRST: Facilitate, Identify, Respect, Support and Training
GRIPP: Guidance for Reporting Involvement of Patients and the Public
HTA: Health technology
IAP2: International Association for Public Participation
ICMJE: International Committee of Medical Journal Editors
LF: Long form
NHC: National Health Council
NICE: National Institute for Health and Care Excellence
NIH: National Institutes of Health
NIHR: National Institute for Health Research
OMERACT: Outcome Measures in Rheumatology conferences
PAO: Patient Advocacy Organization
PBA: Person-Based Approach
PCORI: Patient-Centered Outcomes Research Institute
PFDD: Patient-Focused Drug Development
PFMD: Patient Engagement for Medicines Development
PoPPIE: People and Patient Participation Involvement and Engagement
PPI: Patient and Public Involvement
PPRNs: Patient-Powered Research Networks
PROs: Patient Reported Outcome measures
SF: Short Form
SPOR: Strategy for Patient-Oriented Research
