# Supplementary material for: Patient and public involvement in research: a review of practical resources for young investigators
Source: BMC Rheumatol. 2023 Mar 9;7:2. doi: 10.1186/s41927-023-00327-w (PMC9996937; doi:10.1186/s41927-023-00327-w)
Supplement: Supplementary file 1 — Additional file 1: A summary of web links to various tools and resources for PPI in various stages of research. [file 41927_2023_327_MOESM1_ESM.docx]

**Addition file 1.**

**A summary of web links to various tools and resources for PPI in various stages of research**

| **Links to Tools & Resources** | Descriptors |
| --- | --- |
| **PPI Frameworks, Guidelines, Principles, Toolkits** |  |
| <https://pemsuite.org/How-to-Guides/Early-Discovery.pdf> | Patient Engagement for Medicines Development (PFMD) How-to guide for patient engagement in the early discovery and preclinical phases |
| <http://imi-paradigm.eu/petoolbox/pe-capabilities/> | Innovative Medicines Initiative (IMI) PARADIGM. Patient Engagement Capabilities |
| <https://involvementtoolkit.clinicaltrialsalliance.org.au/toolkit/> | Australian Clinical Trials Alliance (ACTA) Consumer Involvement and Engagement Toolkit |
| <https://www.pcori.org/engagement/engagement-resources> | Patient-Centered Outcomes Research Institute (PCORI) |
| <https://www.invo.org.uk/wp-content/uploads/2014/11/9938_INVOLVE_Briefing_Notes_WEB.pdf> | NIHR INVOLVE Briefing Notes for Researchers |
| <https://nationalhealthcouncil.org/wp-content/uploads/2019/12/PatientEngagement-WhitePaper.pdf> | National Health Council (NHC) patient engagement whitepaper |
| <https://cihr-irsc.gc.ca/e/documents/spor_framework-en.pdf> | The Canadian Institutes of Health Research (CIHR)  Strategy for Patient-Oriented Research - Patient Engagement Framework |
| <https://www.cfhi-fcass.ca/innovations-tools-resources/patient-engagement-resource-hub> | Healthcare Excellence Canada  Innovations, Tools and Resource |
| <https://onlinelibrary.wiley.com/doi/epdf/10.1002/art.22091>  <https://www.ncbi.nlm.nih.gov/pmc/articles/PMC5060802/pdf/HEX-18-489.pdf> | The FIRST (Facilitate, Identify, Respect, Support and Training) model |

| <https://www.thelancet.com/action/showPdf?pii=S0140-6736%2819%2930034-0> | European Society for Clinical and Economic Aspects of Osteoporosis, Osteoarthritis and Musculoskeletal Diseases (ESCEO) and the World Health Organization (WHO) Practical Guidance for Engaging Patients in Health Research |
| --- | --- |
| <https://ard.bmj.com/content/70/5/722.long> | The European League Against Rheumatism (EULAR) |
| <https://www.jrheum.org/content/43/1/187.long> | The Outcome Measures in Rheumatology conferences (OMERACT) |
| https://ctti-clinicaltrials.org/wp-content/uploads/2021/06/CTTI_Patient_Group_Engagement_Recs.pdf | Clinical Trials Transformation Initiative (CTTI) Working with Patient Advocacy Organizations (PAOs) |
| [Patient involvement in the development, regulation and safe use of medicines - CIOMS](https://cioms.ch/publications/product/patient-involvement/) | Council for International Organizations of Medical Sciences (CIOMS) |
| **Authorship, Publications, Good Lay Summary Practice, Dissemination** |  |
| <https://www.icmje.org/recommendations/browse/roles-and-responsibilities/defining-the-role-of-authors-and-contributors.html> | The International Committee of Medical Journal Editors (ICMJE) Defining the Role of Authors and Contributors |
| <https://doi.org/10.1136/bmj.j3453> | GRIPP2 reporting checklists to improve reporting of patient and public involvement in research |
| extension://elhekieabhbkpmcefcoobjddigjcaadp/https://health.ec.europa.eu/system/files/2021-10/glsp_en_0.pdf | Good Lay Summary Practice Guidance |
| <https://www.plainlanguage.gov/media/FederalPLGuidelines.pdf> | USA Federal Plain Language Guidelines 2011 |
| <https://pemsuite.org/How-to-Guides/WG5.pdf> | PFMD Plain language summaries (PLS) of peer-reviewed publications and conference presentations: practical ‘How-To’ Guide for multi-stakeholder co-creation |
| <https://patientsincluded.org/conferences/> | The Patients Included™ conference charter provides conference organizers with information to include patient engagement in conferences. |
| **Patient Engagement Plan, Agreements, Contracts** |  |
| <http://imi-paradigm.eu/petoolbox/contract-templates/> | IMI-PARADIGM  Patient Engagement Agreement Templates-  Consultancy, Speaker, Collaboration, Advisory Board |
| <https://pemsuite.org/legal-and-contractual-tools/> | PFMD - various tools to help you set up and run a patient engagement initiative |
| <https://www.pcori.org/sites/default/files/PCORI-Updated-Engagement-Plan-Template.pdf> | PCORI Patient Engagement Plan |
| <https://www.scpor.ca/patient-engagement-planning-tool> | Strategy for Patient-Oriented Research (SPOR) Patient Engagement Planning Tool |
| **Payment/Remuneration** |  |
| <https://ontariocaregiver.ca/wp-content/uploads/2021/06/Should-money-come-into-it.pdf> | The Change.org Decision Tool; Should money come into it? |
| <https://www.pcori.org/sites/default/files/PCORI-Compensation-Framework-for-Engaged-Research-Partners.pdf> | PCORI Varying Levels of Compensation Model & Framework |
| <https://www.efpia.eu/media/413114/workingtogetherwithpatients_patient-remuneration-principles.pdf> | The European Federation of Pharmaceutical Industries and Associations (EFPIA) Patient Remuneration Principles |
| <https://nationalhealthcouncil.org/wp-content/uploads/2021/06/NHC-_FMV_Patient-Engagement-Compensation_Prinicples-final_.pdf> | The National Health Council (NHC) Principles for Compensating Patients for Patient Engagement Activities |
| <https://nationalhealthcouncil.org/fair-market-value-calculator/> | The NHC Patient engagement fair-market value calculator. |
| <https://pemsuite.org/fmv/> | PFMD - Fair Market Value Principles |
| <https://www.efpia.eu/media/413114/workingtogetherwithpatients_patient-remuneration-principles.pdf> | EFPIA Patient Remuneration Principles |
| <https://www.nihr.ac.uk/documents/centre-for-engagement-and-dissemination-recognition-payments-for-public-contributors/24979> | Centre for engagement and dissemination—recognition payments for public contributors. National Institute for Health Research (NIHR) |
| <https://cihr-irsc.gc.ca/e/51466.html> | CIHR Considerations when paying patient partners in research |
| **Identifying Patients** |  |
| <https://pemsuite.org/How-to-Guides/Questions-to-understand-the-condition-and-therapy-area.pdf> | PFMD Survey to identify patient partners through understanding the condition and therapy area Attachment to Book 1. Section 2.2. Co-developing discussion questions with patients |
| [EUPATIConnect: Bringing EUPATI Patient Experts and researchers together - EUPATI](https://eupati.eu/news/eupaticonnect-bringing-eupati-patient-experts-and-researchers-together/) | The European Patients’ Academy on Therapeutic Innovation (EUPATI) CONNECT |
| <https://imi-paradigm.eu/> | Patient Toolkit, training, |
| <https://www.imi.europa.eu/get-involved/patients/imi-pool-patient-experts> | Europe IMI Patient Pool |
| [Partners \| PatientsLikeMe](https://www.patientslikeme.com/partners) | Patients Like Me |
| [https://www.savvy.coop](https://www.savvy.coop/) | Savvy cooperative- a patient-owned public benefit co-op which facilitates partnership with diagnosed patients |
| [Personalizing Research: Involving, Inviting, and Engaging Patient Researchers \| SpringerLink](https://link.springer.com/chapter/10.1007/978-3-031-07696-1_17) | Dana Lewis Personal Health Informatics publication with suggestions on identifying patient partners & how to help patient-led research |
| **Patient Toolkits, Training** |  |
| <https://www.eu-patient.eu/Projects/eupati2/> | EUPATI Individual Patient Training; The European Patients' Academy (EUPATI) is a pan-European [Innovative Medicines](https://www.ema.europa.eu/en/glossary/innovative-medicine) Initiative project of 33 organizations, led by the European Patients' Forum, with partners from patient organizations, universities and not-for-profit organizations, along with a number of European pharmaceutical companies. It provides scientifically reliable, objective and comprehensive information to patients on medicines research and development. |
| [EURORDIS Open Academy](https://openacademy.eurordis.org/) | The [Open Academy School on Medicines Research & Development](https://openacademy.eurordis.org/) helps prepare patients for advocating for medicines development, equal access to treatments across Europe and ensuring that medical information is clear, accurate and comprehensible. The European Organisation for Rare Diseases (EURORDIS) launched this training program for patient experts in 2008. |
| [e-learning.transmedacademy.eu](https://e-learning.transmedacademy.eu/) | European Research Infrastructure for Translational Medicine (EATRIS) |
| <https://www.ema.europa.eu/en/partners-networks/patients-consumers/training-resources-patients-consumers#training-resources-section> | European Medicines Agency (EMA) Training and resources for patients and consumers |
| <https://learning.pfmd.org/> | PFMD Patient Engagement Training |
| <https://www.pcori.org/engagement/research-fundamentals> | PCORI Research Fundamentals Training |
| <https://involvementtoolkit.clinicaltrialsalliance.org.au/media/z2odqoly/cip-final.pdf> | Australian Clinical Trials Alliance/ACTA Consumer Involvement and Engagement Toolkit |
| <https://wecanadvocate.eu/patients-in-publications/> | Patients in Publications Online Training Course for Patient Advocates |
| **Patient Preference Recommendations, Guidance and Good Research Practices** |  |
| <https://zenodo.org/record/6491042#.Yn6oQujMI2y> | The IMI PREFER (Patient Preferences in Benefit–Risk Assessments during the Medical Product Lifecycle) consortium developed recommendations - Why, when and how to assess and use patient preferences in medical product decision-making |
| <https://www.fda.gov/regulatory-information/search-fda-guidance-documents/patient-focused-drug-development-collecting-comprehensive-and-representative-input> | FDA Guidance – PFDD Collecting Comprehensive and Representative Input |
| J.F.P. Bridges, et al., “Conjoint analysis applications in health—a checklist: a report of the ISPOR Good Research Practices for Conjoint Analysis Task Force,” Value in Health, 403-13 (2011).  F.R. Johnson, et al., “Constructing experimental designs for discrete-choice experiments: Report of the ISPOR conjoint analysis experimental design good research practices task force,” Value in Health, 3-13 (2013).  A.B. Huber, J. González, C.G.M. Groothuis-Oudshoorn, T. Prior, D.A. Marshall, C. Cunningham, M.J. IJzerman, J.F.P. Bridges, “Statistical Methods for the Analysis of Discrete Choice Experiments: A Report of the ISPOR Conjoint Analysis Good Research Practices Task Force,” Value in Health, available online: http://dx.doi.org/10.1016/j.jval.2016.04.004, (2016). | International Society for Pharmacoeconomics and Outcomes Research (ISPOR) published a set of good research practices for preference-based methods. |
| **FDA Guidances relevant to PPI** |  |
| <https://www.fda.gov/regulatory-information/search-fda-guidance-documents/patient-focused-drug-development-methods-identify-what-important-patients> | FDA Guidance- PFDD Methods to Identify What is Important to Patients |
| <https://www.fda.gov/regulatory-information/search-fda-guidance-documents/patient-focused-drug-development-selecting-developing-or-modifying-fit-purpose-clinical-outcome> | Draft FDA Guidance- PFDD Selecting, Developing, or Modifying Fit for Purpose Clinical Outcomes |
| <https://www.fda.gov/regulatory-information/search-fda-guidance-documents/principles-selecting-developing-modifying-and-adapting-patient-reported-outcome-instruments-use> | FDA Guidance- Principles for Selecting, Developing, Modifying, or Adapting Patient Reported Outcome Instruments |
| <https://www.fda.gov/regulatory-information/search-fda-guidance-documents/patient-engagement-design-and-conduct-medical-device-clinical-studies> | Final Good Clinical Practice |
| <https://www.fda.gov/regulatory-information/search-fda-guidance-documents/developing-and-submitting-proposed-draft-guidance-relating-patient-experience-data> | Draft FDA Guidance- Developing and Submitting Patient Experience Data |
| <https://www.fda.gov/regulatory-information/search-fda-guidance-documents/patient-focused-drug-development-collecting-comprehensive-and-representative-input> | FDA Guidance- PFDD, Collecting Comprehensive & Representative Input |
| <https://www.fda.gov/regulatory-information/search-fda-guidance-documents/patient-reported-outcome-measures-use-medical-product-development-support-labeling-claims> | FDA Guidance- Patient Reported Outcomes |
| **PPI Quality Improvement** |  |
| <https://patientfocusedmedicine.org/peqg/patient-engagement-quality-guidance.pdf> | PFMD Patient Engagement Quality Guidance |
| **Topics not covered here, yet essential for enduring PPI** |  |
| [PED Navigator - PEM Suite](https://pemsuite.org/ped-navigator/) | Patient Engagement Open Forum (PEOF) Global Patient Experience Data Navigator |
| <https://www.atsdr.cdc.gov/communityengagement/pdf/PCE_Report_508_FINAL.pdf> | CDC Principles of Community Engagement |
| <https://iap2content.s3-ap-southeast-2.amazonaws.com/marketing/Resources/Tools+and+Templates/Valuing-Better-Engagement-Economic-Framework_2015.pdf> | The International Association for Public Participation (IAP2) PWC (Australia) - valuing better engagement proudly supported by: an economic framework to quantify the value of stakeholder engagement for infrastructure delivery |
| <https://ukhomeoffice.github.io/accessibility-posters/> | UK Designing accessible services |
| <https://www.transceleratebiopharmainc.com/assets/patient-technology-implementation-framework/> | Transcelerate  Patient Technology Implementation Framework |
| <https://www.durham.ac.uk/research/institutes-and-centres/social-justice-community-action/toolkits/> | Community based research project |
